# A TiO_2_/C catalyst having biomimetic channels and extremely low Pt loading for formaldehyde oxidation†

**DOI:** 10.1039/c8ra10314c

**Published:** 2019-01-29

**Authors:** Wei Liu, Yutao Gong, Xueping Li, Cai-wu Luo, Congmin Liu, Zi-sheng Chao

**Affiliations:** State Key Laboratory of Chemo/Biosensing and Chemometrics, College of Chemistry and Chemical Engineering, Hunan University Changsha 410082 China zschao@yahoo.com; School of Chemical & Biomolecular Engineering and RBI, Georgia Institute of Technology 500 10th Street N.W. Atlanta GA 30332 USA; College of Materials Science and Engineering, Changsha University of Science and Technology Changsha Hunan 410114 China; National Institute of Clean-and-Low-Carbon Energy Beijing 102211 China

## Abstract

This study presents a TiO_2_/C hybrid material with biomimetic channels fabricated using a wood template. Repeated impregnations of pretreated wood chips in a Ti precursor were conducted, followed by calcination at 400–600 °C for 4 hours under a nitrogen atmosphere. The generated TiO_2_ nanocrystals were homogenously distributed inside a porous carbon framework. With an extremely low Pt catalyst loading (0.04–0.1 wt%), the obtained porous catalyst could effectively oxidize formaldehyde to CO_2_ and H_2_O even under room temperature (conv. ∼100%). Wood acted as both a structural template and reduction agent for Pt catalyst generation in sintering. Therefore, no post H_2_ reduction treatment for catalyst activation was required. The hierarchal channel structures, including 2–10 nm mesopores and 20 μm diameter channels, could be controlled by calcination temperature and atmosphere, which was confirmed by SEM and BET characterizations. Based on the abundant availability of wood templates and reduced cost for low Pt loading, this preparation method shows great potential for large-scale applications.

## Abstract 2

This study presents a TiO_2_/C hybrid material with biomimetic channels fabricated using a wood template.

## Graphical abstract

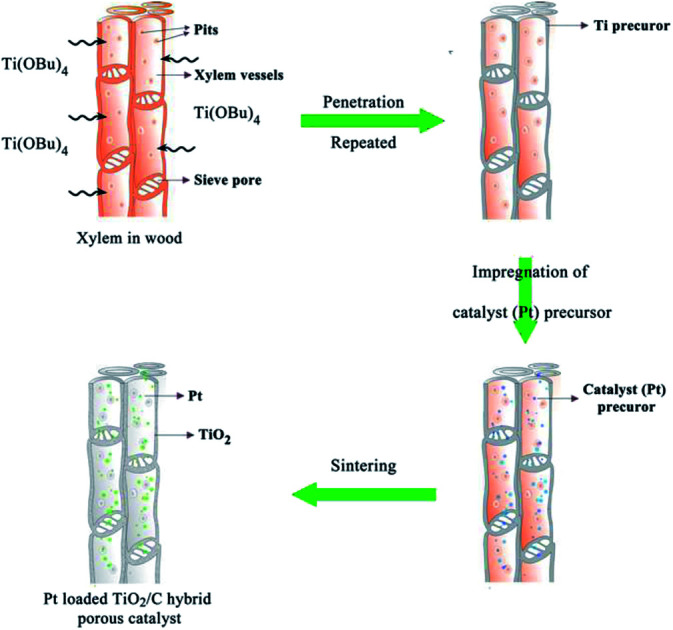

Formaldehyde (HCHO) is an important chemical feedstock that is widely used in the production of industrial resins, polymers and coating materials. However, formaldehyde is highly toxic and volatile, and it has been listed as a human carcinogen group I by the International Agency for Research on Cancer (IARC).^1^ Formaldehyde is slowly emitted from building and furnishing materials, which can cause serious health problems. Developing an efficient and feasible catalyst for the removal of formaldehyde pollutant from indoor environment has received a growing concern recently.

Inorganic porous materials are good candidates for application in the removal of formaldehyde either by physical absorption or chemical catalytic oxidation.^2,3^ Inorganic porous materials have multi-shapes of pore structures and large surface areas, which allow formaldehyde molecules access into holes. Moreover, the inorganic framework of the material has high thermal stability, suggesting that the material can be thermally regenerated and reused for many times. Many inorganic porous material-based formaldehyde absorbents, such as active carbon,^4^ porous Al_2_O_3_ (ref. 5) and zeolite,^6^ have been investigated. For example, different porous Al_2_O_3_ materials were prepared by using the template method, which showed broad pore size distribution and good formaldehyde adsorption–desorption behavior.^7,8^ However, the porous inorganic adsorbents have limited adsorption capacities, resulting in only a short effective time.

Formaldehyde oxidation over porous material-supported catalysts is a promising technology because formaldehyde can be continuously oxidized to CO_2_ and H_2_O.^9–11^ The porous structure can facilitate fast diffusion and mass transport of formaldehyde and products. Furthermore, the high surface area of the porous material provides a large number of active sites for formaldehyde adsorption and catalyst loading. Many catalyst-loaded porous materials or porous transition metal oxides were prepared and displayed excellent formaldehyde oxidation performance; some examples include Pt on ZSM-5 and NaY zeolites,^12,13^ mesoporous Au, Pt or Pd/CeO_2_,^14–16^ VO_*x*_/MCM,^17^ macro-mesoporous Pt/γ-Al_2_O_3_,^18^ Pd or Pt/TiO_2_,^19–24^ Pt/mesoporous ferrihydrite^25^ and porous MnO_2_.^26,27^ Various organic templates were used in the preparation of TiO_2_ with desired porosity, such as surfactants,^28,29^ block copolymers^30–32^ and even small organic molecules including salicylic acid and aspartic acid.^33^ However, these organic templates generally have a high price, which hinders their potential for large-scale applications. Another issue is that the noble metal catalyst loading is still high (1–2.5%) to reach effective formaldehyde oxidation.

Biomass materials have low cost and natural hierarchal structures in the range from millimeters to nanometers,^34^ which can be used as natural templates for porous material fabrication. TiO_2_ with a porous structure has been prepared by bio-templating of naturally grown biomass materials, such as wood and bamboo;^22,35^ however, there is no reported example on the use of the material as a catalyst in reactions such as organic oxidation. Herein, we presented a wood templated TiO_2_/C material having biomimetic channel structures. The obtained material was different from the supported catalysts or core–shell structure materials, in which the catalyst layers are only coated inside or outside the porous structure. Wood templated TiO_2_/C exhibited a hybrid structure; this resulted in the generation of TiO_2_ nanocrystals, which were homogenously distributed inside the porous carbon framework because the Ti precursor penetrated into wood tissues before catalyst sintering. With an extremely low loading of Pt, this type of hierarchal structured material showed highly effective formaldehyde oxidation behavior even under room temperature.

## Results and discussion

**Fig. 1:**
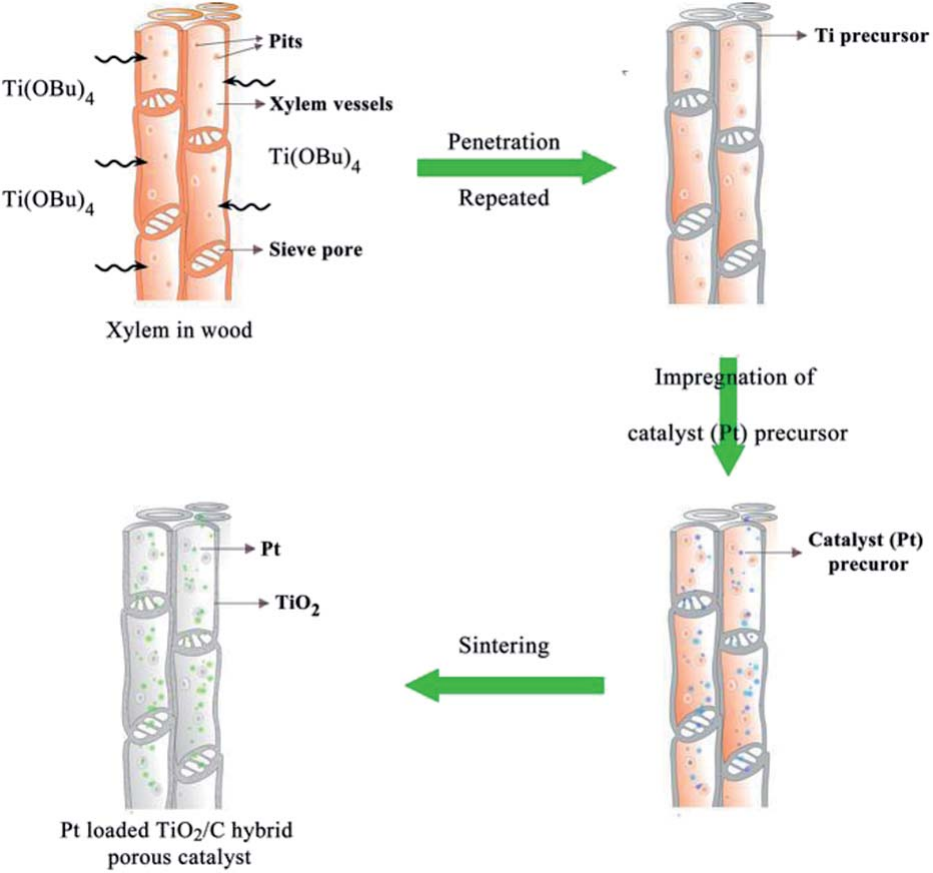
Schematic illustration of wood templated biomimetic TiO_2_/C hybrid material preparation.

Fig. 1 shows the preparation process and schematic illustration of the obtained TiO_2_/C hybrid porous structure. Simple impregnation of wood chips in the organic titanium precursor and the following drying process were repeated alternately. After the Ti precursor penetrated into wood tissues, the Pt precursor also infiltrated into wood and then was calcinated under a controlled temperature and atmosphere.

**Fig. 2:**
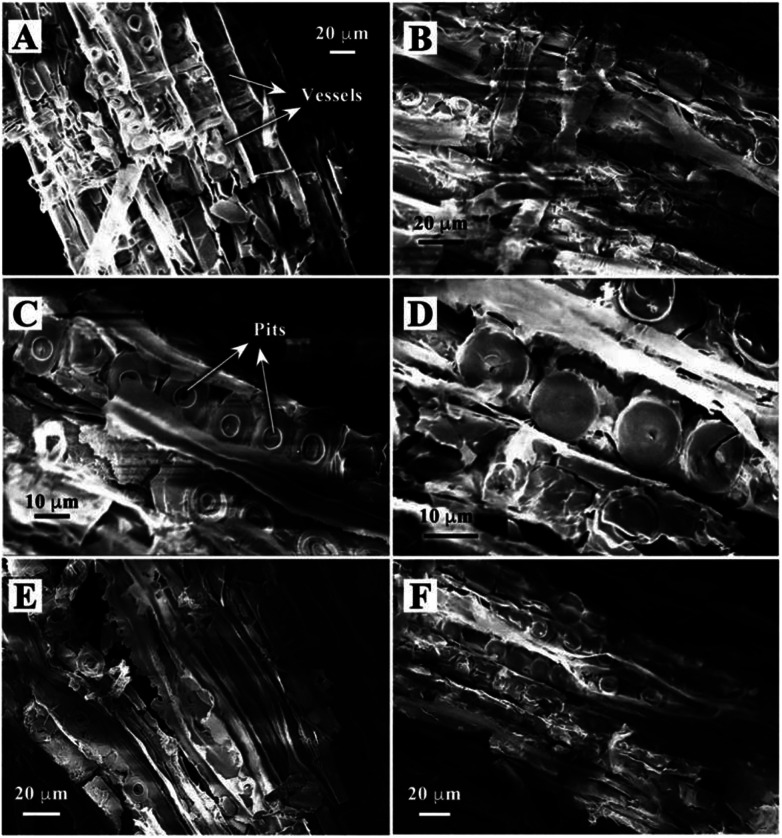
SEM images of TiO_2_/C material containing biomimetic channels prepared by a wood template after sintering at 400 °C (A–D), 500 °C (E) and 600 °C (F).

The morphology of biomimetic channel-containing TiO_2_/C material prepared by the wood template is shown in Fig. 2, showing multi-scaled hierarchal structures. As shown in Fig. 2A, the channel structure is formed after sintering at 400 °C. This could be produced by the xylem vessels in wood. During the impregnation process, the organic titanium precursor Ti(OBu)_4_ diffused into the xylem vessels and tissues or was coated on the surface of vessel walls (Fig. 1). Furthermore, pre-formed TiO_2_ sol could also be filled into the vessels of wood; after solvents evaporated, the TiO_2_ sol was fixed on the vessel walls. The TiO_2_ precursor layer could be increased by repeating the impregnation and drying processes alternatively. With sintering at a high temperature, the organic wood components, including hemicellulose, cellulose and lignin, were converted to porous carbon. The TiO_2_ precursor was transformed into TiO_2_ and the whole material maintained the physical structure of the wood template. The formed TiO_2_/C channels exhibited diameter of *ca.* 20 μm, which was the same as the size of xylem vessels in wood. Some pits were observed in the TiO_2_ channels. Pits are cavities located on wood cell walls, which are essential components for water-transport system in wood.^36^ The pitted structure was maintained perfectly through the impregnation method after sintering, which increased the porosity and helped improve the catalytic performance over the obtained material. As shown in the magnified images (Fig. 2C and D), the pits exhibit a cylindrical shape with diameter of *ca.* 10 μm.

Different samples were prepared under the sintering temperatures of 400, 500 and 600 °C in this study. The samples showed similar structures, *i.e.*, hierarchical channels. The obtained morphologies under sintering at 500 and 600 °C are shown in Fig. 2E and F. The SEM images verify the successful preparation of a porous TiO_2_ material templated by wood.

Before sintering, the catalyst (Pt) precursor was loaded into the channel structure of the material. The loading process was similar to the preparation of a TiO_2_ layer, but a solution of H_2_PtCl_6_ dissolved in isopropanol was used. The desired amount of catalyst could be reached by repeating the impregnation and drying processes. The advantage of the impregnation method for catalyst loading is the homogenous dispersion of the catalyst. In order to investigate the chemical composition, elemental mapping was performed using energy-dispersive spectrometry (EDS) (shown in ESI, Fig. S1.†). For the sample calcined at 400 °C, the results showed that the TiO_2_ component included 25.3% and 74.5% of carbon and 0.1% of Pt loading amount. The exact Pt contents in the prepared catalysts were measured by an inductively coupled plasma mass spectrometer (ICP-MS), showing that the samples calcined at 400, 500 and 600 °C contained 0.092%, 0.095% and 0.098% weights of Pt, respectively. In addition, the catalysts with different amounts of Pt loading were studied, and Pt loadings were found to be 0.063% and 0.041% (shown in ESI Table S1†).

**Fig. 3:**
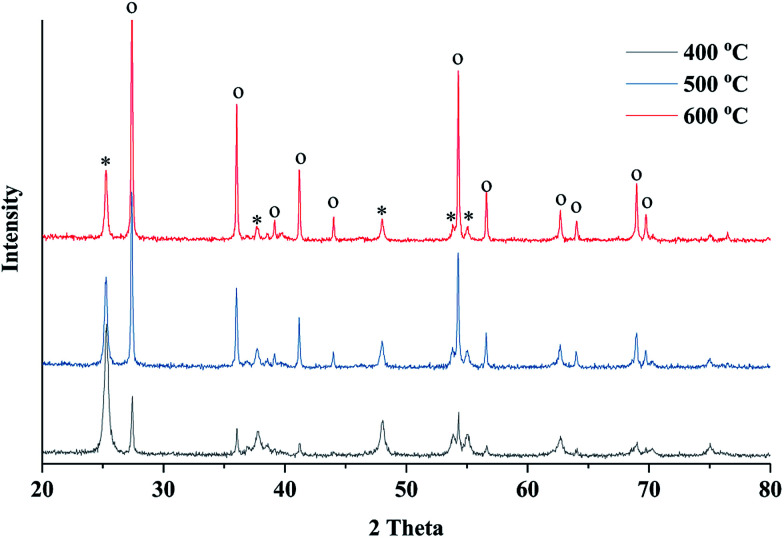
XRD patterns of the obtained TiO_2_ material sintered at 400, 500 and 600 °C for 4 hours (o denotes rutile phase and * denotes anatase phase).

X-ray diffraction (XRD) was employed to study the crystalline phase of prepared porous TiO_2_/C materials. Fig. 3 shows the XRD patterns of different samples calcined at 400, 500 and 600 °C. As the calcination temperature increased, the intensity of XRD peak increased, indicating the increase in crystallinity of TiO_2_ compared to the amorphous phase. The XRD peaks could be identified by comparing with the standard diffraction peaks of anatase and rutile TiO_2_, showing that the samples contained both anatase and rutile phases of TiO_2_. The XRD peaks at 2*θ* = 25.25° (101) and 2*θ* = 48.03° (200) verified the formation of the anatase phase, while the peaks at 2*θ* = 27.42° (110) and 2*θ* = 36.08° (101) indicated the presence of the rutile phase.^37,38^ Significant phase transformation from anatase to rutile was observed under a high sintering temperature. The ratio of anatase to rutile can be estimated by the intensity of characteristic diffraction peaks of both phases. According to the Spurr–Meyers equation, the weight percentages of anatase (*X*_A_) and rutile (*X*_R_) can be calculated as follows:^39,40^

1 *X*_A_ = 1/(1 + 1.265*I*_R_/*I*_A_) × 100%

2 *X*_R_ = 1/(1 + 0.8*I*_A_/*I*_R_) × 100%

Here, *I*_A_ is the anatase phase intensity at 2*θ* = 25.25°; *I*_R_ is the intensity of rutile peak at 2*θ* = 27.42°.

The calculation results showed that the anatase phase accounted for 63.5% in the sample sintered at 400 °C, but this value significantly decreased to 16.8% when a high sintering temperature of 600 °C was applied (Table 1). The average crystalline size of obtained TiO_2_ was calculated by Debye–Scherrer equation, indicating that the crystal size increased at a high calcination temperature.

**Table 1:** Weight percentage (*X*) and crystalline size (*D*) of anatase (A) and rutile (R) phases in prepared TiO_2_ samples

Temp. (°C)	*X*_A_ (%)	*X*_R_ (%)	*D*_A_ (nm)	*D*_R_ (nm)
400	63.5	36.2	20.3	119.8
500	28.7	71.0	27.0	120.5
600	16.8	83.0	30.5	131.7

To investigate the phase transition behavior with the wood template, TiO_2_ samples were prepared using the sol–gel method without wood template but with the same calcination conditions. The XRD characterization showed a high content ratio of anatase phase in the TiO_2_ samples prepared using the sol–gel method (shown in ESI, Fig. S2.†). However, in wood templated TiO_2_ preparation, all samples showed anatase and rutile mixed phases. These results indicated that the wood template actually promoted the anatase-to-rutile phase transition in TiO_2_ powders because the transformation behavior can be affected by many factors such as impurities in wood, grain sizes of formed TiO_2_ crystals, preparation methods and annealing conditions.

**Fig. 4:**
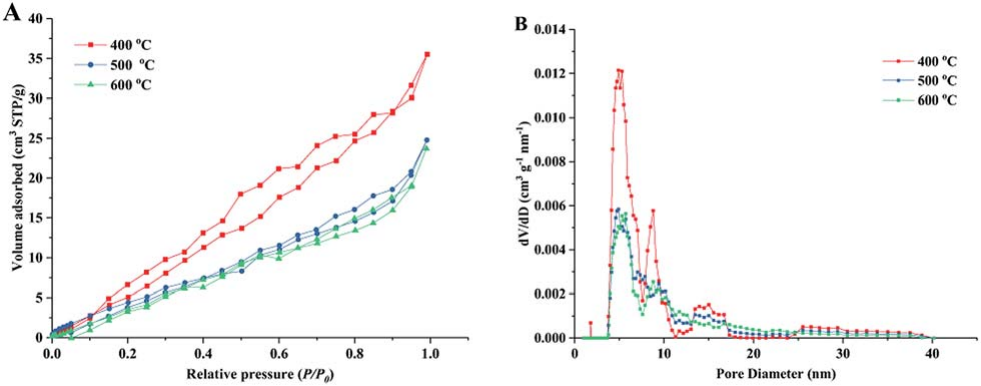
Nitrogen adsorption/desorption isotherm curves (A) and corresponding pore-size distribution curves (B) of prepared TiO_2_ materials under different sintering temperatures.

Fig. 4 shows the nitrogen adsorption isotherms and pore size distribution curves of the obtained porous TiO_2_ material templated by wood. The isotherm curves of all samples can be classified as type-IV isotherms based on the Brunauer–Deming–Deming–Teller classification,^41^ indicating the mesoporous structure in the obtained TiO_2_/C material. The isotherms show hysteresis loops that have an H3-type shape,^42^ suggesting the presence of a layered structure with slit-like pores (multi-channel structures in this material). These results were consistent with the structure observed using SEM. The specific surface area was 41.5 m^2^ g^−1^ for the sample sintered at 400 °C and the value slightly decreased with the high sintering temperatures of 500 and 600 °C. The pore volumes of the obtained TiO_2_/C material were in the range of 0.055–0.033 cm^3^ g^−1^. The specific surface areas and pore volumes of obtained samples in this study were similar to those of previously reported biomass templated materials.^35,43^ The corresponding pore size distribution curves show that the wood templated TiO_2_ materials have meso-pores with width of 2–10 nm (shown in ESI, Table S2.†). It should be noted that some pores exhibited large size of 12–18 nm. The large-sized pores could be formed by wood template removal during sintering. The micro-sized channel and nano-sized pore structure formed a hierarchal channel system in the TiO_2_ material, which largely improved the catalytic behavior in formaldehyde oxidation.

**Fig. 5:**
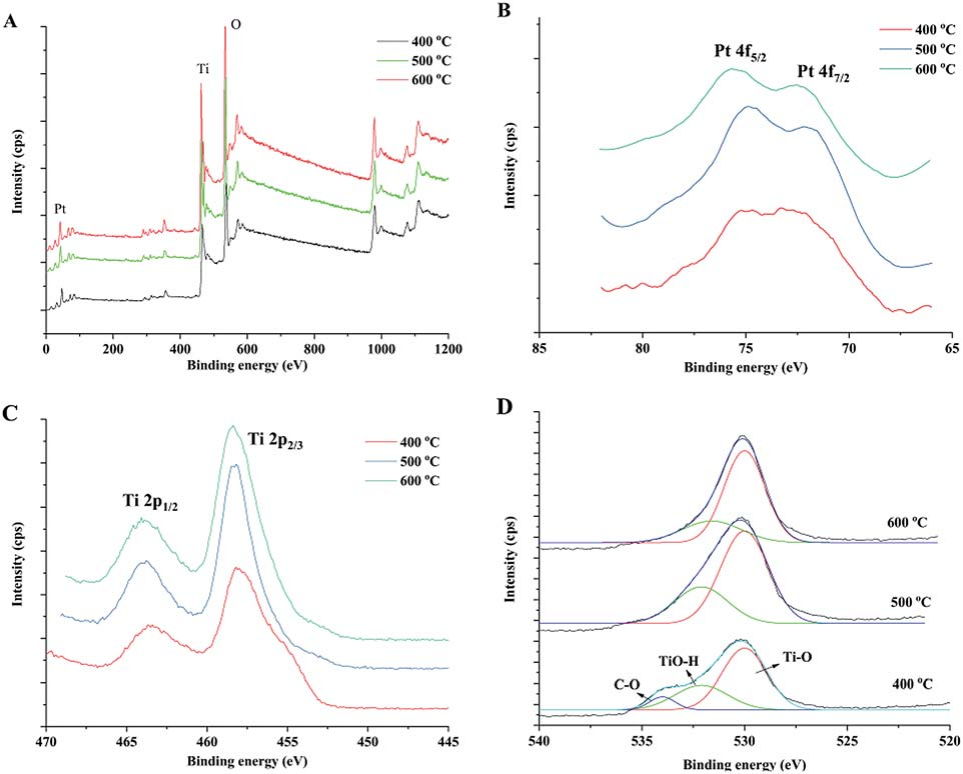
XPS survey spectra (A) of the obtained catalysts and high resolution spectra of Pt 4f (B), Ti 2p (C) and O 1s (D) of prepared TiO_2_ samples under different sintering temperatures.

**Fig. 6:**
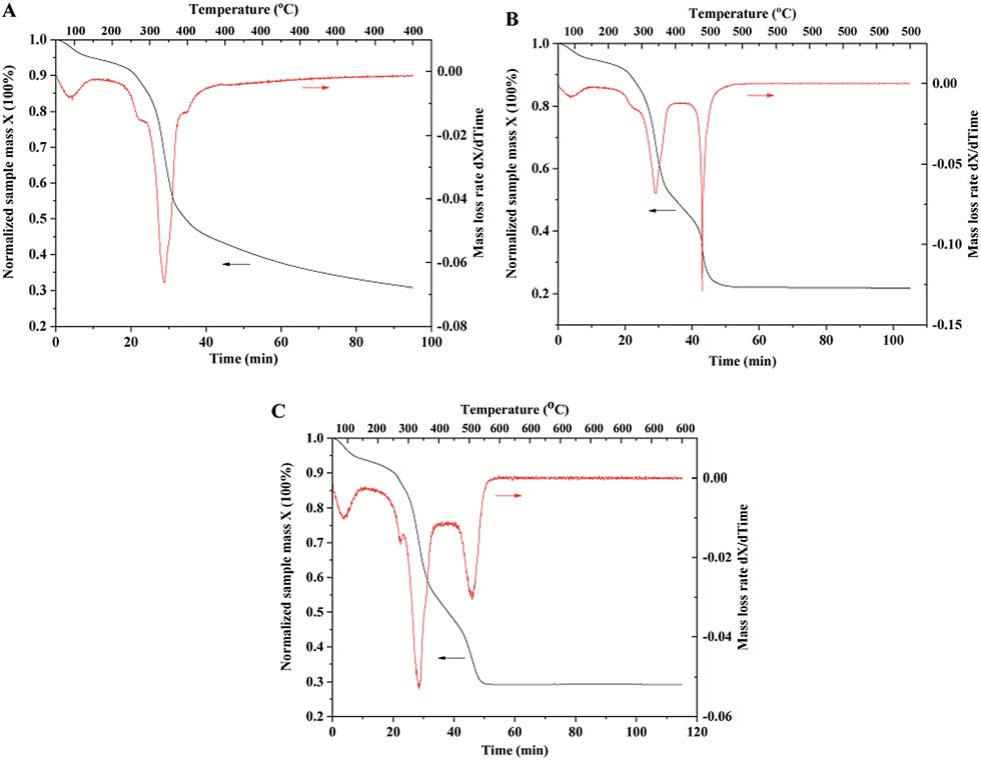
TGA curves of wood samples impregnated with TiO_2_ and catalyst precursors. (A) Constant temperature at 400 °C for 1 hour under air atmosphere; (B) 500 °C; (C) 600 °C.

The chemical status of the elements in the samples was analysed by X-ray photoelectron spectroscopy (XPS). XPS survey spectra (Fig. 5A) of prepared samples indicate the presence of C, Ti, O and Pt and the corresponding photoelectron peaks are located at binding energies of 284.8 (C 1s), 458.8 (Ti 2p), 529.8 (O 1s) and 70.6 eV (Pt 4f). Fig. 6 shows the high-resolution XPS spectra of Pt 4f, Ti 2p and O 1s of the prepared samples. The peaks at 71.1 and 74.3 eV could be attributed to Pt 4f_7/2_ and Pt 4f_5/2_ bands for metallic Pt (Pt^0^), respectively.^44,45^ For Pt^2+^ and Pt^4+^, the binding energies were reported as *ca.* 73.0 and 74.7 eV for 4f_7/2_ and 76.4 and 78.1 eV for 4f_5/2_.^46^ The Pt 4f XPS spectra verified the reduction of Pt during the sintering process by wood because there was no post reduction treatment by H_2_ gas in this study. Wood acted as both a structural template and reduction agent of Pt catalyst in the preparation process. The Ti 2p spectra are shown in Fig. 5C. The peaks at *ca.* 464.0 and 458.2 eV were ascribed to Ti 2p_1/2_ and Ti 2p_3/2_ of Ti^4+^ in TiO_2_, respectively. The O 1s spectra are shown in Fig. 5D, which include many overlapped peaks. The fitting peak at *ca.* 530 eV represents the binding energy of O 1s of TiO_2_ lattice oxygen (Ti–O–Ti).^20,45^ A significant shoulder peak at *ca.* 533 eV appeared beside the Ti–O–Ti peak in the spectrum of the sample sintered at 400 °C. This shoulder peak included two fitting peaks: one at 531.6 eV, which can be ascribed to the oxygen in Ti–OH group on the TiO_2_ surface, and the other at 533.8 eV, which can be ascribed to organic oxygen in C–O bonds.^47^ These results indicated that there is a small amount of organic residues in the sample after sintering at 400 °C in air for 4 hours. The C–O peak disappeared in the spectra of the samples sintered at 500 and 600 °C, which indicated that the organic components of wood were completely oxidized at high sintering temperatures. The Ti–OH peak also reduced with increase in the sintering temperature. The Ti–OH group, which was certificated to be active in the oxidation of formaldehyde, could be formed by the adsorption of oxygen or H_2_O molecules on the surface of TiO_2_. Therefore, the sample that has a large ratio of Ti–OH groups could have high catalytic activity.

In order to investigate mass changes during the sintering process, thermogravimetric analysis (TGA) was performed under air atmosphere. The wood samples after the impregnation of TiO_2_ and Pt precursors were used for TGA measurements. With the increase in temperature, TiO_2_ and catalyst precursors were transformed into crystalline and metallic forms, and wood was decomposed to carbon. The TGA curve with the temperature up to 400 °C is shown in Fig. 6A. The sample mass continuously decreased with increasing temperature and constant temperature sintering. The mass loss rate curve shows that the temperature at the highest decomposition rate of wood is 350 °C. However, the mass loss rate curve with the temperature up to 500 °C indicates two highest decomposition rate temperatures of 350 and 500 °C, as shown in Fig. 6B. The possible reason is that wood could be pyrolyzed and carbonized under a low temperature of 350 °C and oxidized at a high temperature of 500 °C. Compared with the TGA curve of the original wood template, the result showed that the highest decomposition rate temperatures of wood chips were 350 and 380 °C (shown in ESI, Fig. S3.†). It should be noted that mass was continuously lost at the constant temperature of 500 °C (shown in the curve in Fig. 6B). The organic residues could be completely oxidized if there was sufficient reaction time. The TGA curve with the temperature up to 600 °C has similar result compared with the curve of 500 °C. The sample mass was stable after 1 hour sintering at 500 or 600 °C, indicating complete removal of wood template.

**Fig. 7:**
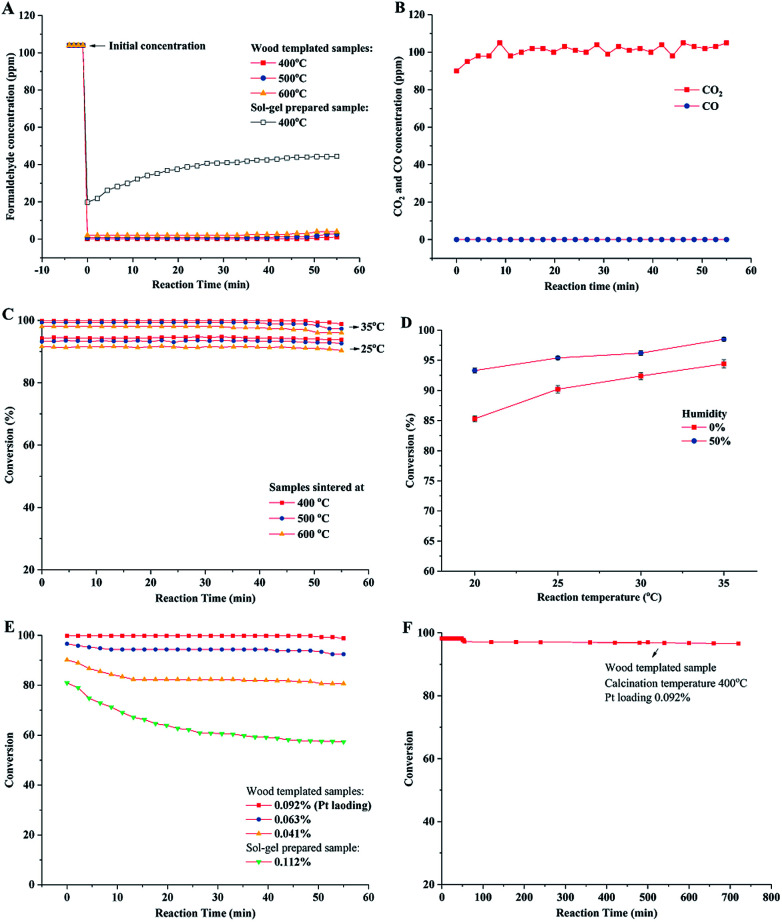
Catalytic oxidation of formaldehyde over prepared porous TiO_2_ catalyst material. (A) The HCOH concentration in upstream and downstream of reactor bed by using different prepared catalysts. (B) The CO_2_ and CO measurements in the reaction of wood templated catalyst (0.092% Pt content, calcined at 400 °C). (C) Performances of catalysts with 400–600 °C calcination temperatures. (D) Performances of catalysts (0.092% Pt content, calcined at 400 °C) under different reaction temperatures and air humidities. (E) Conversions of catalysts with different Pt loading amounts. (F) Long time durability test.

The catalytic behavior of the prepared porous TiO_2_ material was evaluated in a fixed bed reactor. Gaseous HCHO and humid air gas (water vapor 50%) were introduced into the reactor tube, and the HCHO concentration was measured at the outlet of the reactor. The concentrations of HCHO in the stream flowing in and out of the reactor were measured. As shown in Fig. 7A, the HCHO concentration significantly decreases from the initial 104 ppm to *ca.* 0.2–0.5 ppm at the reaction temperature of 35 °C. These results showed high catalytic oxidation performance of formaldehyde over the catalyst with a low Pt loading (0.09%). Anatase phase TiO_2_ was prepared by the sol–gel method without a wood template. The catalytic performance evaluation showed that 44 ppm of HCHO still remained in the downstream of catalyst bed, which indicated low conversion of formaldehyde oxidation. The high catalytic performance of wood templated porous TiO_2_ can be ascribed to the channelled micro–nano hierarchal porous structure and the synergism of anatase–rutile mixed phases. The chemical analysis of oxidation products in the out stream was conducted. It verified that HCHO was completely oxidized to CO_2_ because no CO was detected, as shown in Fig. 7B.

The conversions of formaldehyde oxidation reaction on different prepared catalysts under different reaction temperatures (20–35 °C) and humidities are summarized in Fig. 7C and D. The results indicated that the wood templated TiO_2_ catalyst materials have high catalytic performances in our test time. The sample sintered at 400 °C exhibited higher formaldehyde oxidation conversion than the samples sintered at 500 and 600 °C. This result could be explained by the crystalline phase of TiO_2_ and the amount of hydroxyl groups (Ti–OH) on the surface of the TiO_2_ material. Previous studies show that anatase TiO_2_ has higher catalytic activity than rutile TiO_2_.^44,45,48^ Therefore, the sample sintered at a low temperature exhibited a high content of anatase phase, resulting in high catalytic activity. In addition, the high amount of surface hydroxyl groups led to high oxidation conversion of formaldehyde, which was verified by reported studies.^48,49^ The high Ti–OH ratio confirmed by XPS for the sample sintered at 400 °C also improved the catalytic performance of formaldehyde oxidation. The formaldehyde oxidation performance was related to the humidity of air (Fig. 7D). When dry air was introduced in the oxidation reaction, the conversion slightly decreased. This was because the moisture in air could be adsorbed and reacted with the surface of TiO_2_, resulting in increase in the concentration of hydroxyl groups on the catalyst surface.^50^

The catalysts with different Pt loading amounts were also evaluated in formaldehyde oxidation in a fixed bed reactor (Fig. 7E). The catalyst with 0.092% Pt content showed the highest oxidation conversion (close to 100%). By slightly decreasing the Pt loading amount to 0.063%, the catalyst still showed high performance with the conversion higher than 95%. However, the conversion decreased to around 85% when 0.041% Pt was loaded. Compared with the performance of the sol–gel catalyst without a wood template, the conversion was as low as 60%. The durability of wood templated catalyst was investigated as well. As shown in Fig. 7F, the porous TiO_2_ catalyst calcined at 400 °C maintains high performance in a long test time of 12 hours, showing only 2% decrease in conversion.

**Fig. 8:**
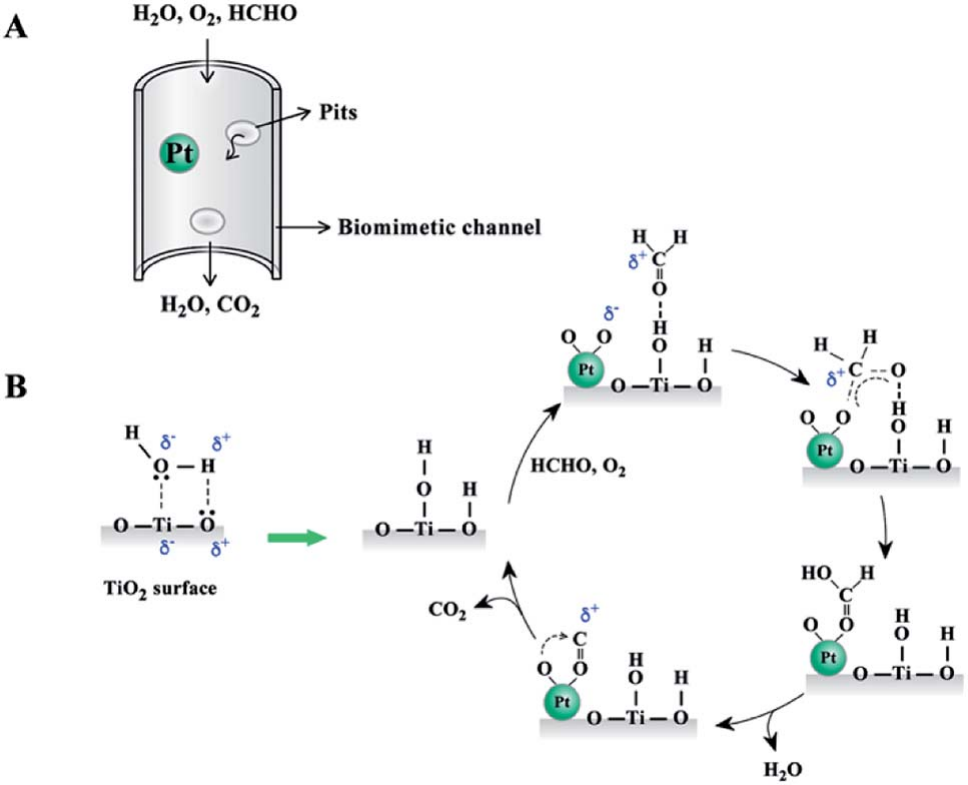
Schematic illustration of reactant and product diffusion in wood templated catalyst (A) and possible mechanism diagram for HCHO oxidation (B).

Fig. 8 illustrates the reaction process between moisture and TiO_2_ surface. The formed hydroxyl groups were active sites for formaldehyde adsorption. The oxidization reaction started with the adsorption of formaldehyde on the TiO_2_ surface and oxygen on the Pt catalyst surface. The absorbed formaldehyde molecules were then transformed to formic acid over the Pt catalyst surface. With the elimination of H_2_O and CO_2_, the catalyst surface could be restored to the initial state, as shown in Fig. 8B. The wood templated channel structure largely improved the reactant and product diffusion and catalytic performance. Therefore, this prepared biomimetic TiO_2_ material showed good catalytic performance even at an extremely low Pt catalyst loading.

## Conclusions

TiO_2_ catalyst materials containing biomimetic channels were successfully prepared by a wood template. SEM images verified the hierarchal channel structure (width *ca.* 20 μm) formed by xylem vessels in wood. BET analysis showed that the prepared TiO_2_ material exhibited 2–10 nm mesopores, which could be formed by small structures in wood-like pits. XRD showed that the prepared TiO_2_ is a mixture of anatase and rutile phases. XPS certified the presence of metallic Pt, which is the active component in the oxidation of formaldehyde. The results of catalytic performance evaluation showed that the prepared TiO_2_ catalysts have high oxidation conversion of formaldehyde.

## Experimental section

### Material

Birch wood chips were supplied by timber trading market. Isopropanol (i-PrOH), benzene (C_6_H_6_), titanium butoxide (Ti[O(CH_2_)_3_CH_3_]_4_) and chloroplatinic acid (H_2_PtCl_6_) were purchased from Alfa Aesar.

### Fabrication method

Birch wood was cut into chips with size of 4 mm × 2 mm × 1 mm. The wood chips were pre-treated with mixed organic solvent (volume of benzene to absolute ethanol was 2 : 1) under reflux for 6 hours to remove the extractives in wood. After solvent was evaporated at 80 °C, the wood chips were washed with deionized water at 80 °C for several times and then dried in oven overnight.

The Ti precursor was prepared by partial hydrolysis of titanium butoxide. Titanium butoxide was dissolved in isopropanol to form 20 wt% solution with stirring (noted as solution A). Water (2 wt%)–isopropanol solution (10 mL) was used as solution B, which was slowly added to 10 mL solution A with stirring. After aging for 1 hour with stirring, the clear and transparent Ti precursor solution was obtained. Pre-treated wood chips were impregnated in the precursor solution under flux for 4 hours and then removed from the solution and dried at 120 °C for 2 hours. This process was repeated until the weight of wood chips increased by 20%.

Catalyst precursor solution was obtained by dissolving chloroplatinic acid (H_2_PtCl_6_) in isopropanol (final H_2_PtCl_6_ concentration, 0.1 wt%). The wood chips after Ti precursor solution treatment were impregnated in catalyst precursor solution at 50 °C for 2 hours with stirring and then dried in an oven overnight. With repeated impregnation and drying processes, different amounts of Pt catalyst loadings could be achieved. Finally, the obtained wood chips were sintered in N_2_ flow. Different samples were prepared at the sintering temperatures of 400, 500 and 600 °C with the heating rate 10 °C min^−1^. Fig. 1 shows the synthesis process of wood templated TiO_2_ catalyst material.

### Characterizations

The amount of loaded Pt was measured by an inductively coupled plasma mass spectrometer (ICP-MS, NexIon, Perkin-Elmer). Before measurement, the samples were dissolved in *aqua regia* (HNO_3_ : HCl = 1 : 3 v/v) with the heating of microwave oven. After cooling, HF solution was added and mineralized to obtain a clear solution. The morphology of obtained porous TiO_2_ catalyst material was characterized by scanning electron microscopy (SEM, Zeiss Ultra60 FE-SEM instrument) coupled with INCA 200 X-Sight de Oxford instruments for energy-dispersive X-ray spectroscopy (EDS) analysis. The formed TiO_2_ crystalline phase was verified by powder X-ray diffraction (PXRD). PXRD was conducted using a PANalytical X'Pert X-ray diffractometer coupled with X'Celerator detector and Cu Kα (*λ* = 1.5418 Å). X-ray photoelectron spectroscopy (XPS) analysis was performed with a Thermo K-Alpha instrument equipped with monochromatic AlKα radiation at 1486.6 eV X-ray source. N_2_ physisorption isotherms were collected at 77 K on a Quadrasorb system from Quantachrome Instruments. Before the measurements, the sample (∼50 mg) was degassed under dynamic vacuum at 150 °C for 18 hours. TGA analysis results were obtained using an STA 6000 Simultaneous Thermal Analyzer (PerkinElmer) with the heating rate of 10 °C min^−1^ and air flow of 10 mL min^−1^.

### Catalytic formaldehyde oxidation test

The catalytic formaldehyde oxidation test was performed in a fixed-bed quartz flow reactor with a gas mixture of air containing 100 ppm HCHO at a low rate 50 cm^3^ min^−1^. The humidity of air gas was controlled at 50% by bubbling through water. The concentrations of HCHO, CO and CO_2_ were analyzed by gas chromatography (GC).

The conversion of HCHO was calculated according to the following equation:
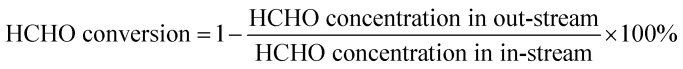


## Conflicts of interest

There are no conflicts to declare.

## Supplementary Material

RA-009-C8RA10314C-s001
