# Supplementary material for: A TiO2/C catalyst having biomimetic channels and extremely low Pt loading for formaldehyde oxidation
Source: RSC Adv. 2019 Jan 29;9(7):3965–71. doi: 10.1039/c8ra10314c (PMC9060426; doi:10.1039/c8ra10314c)
Supplement: RA-009-C8RA10314C-s001 [file RA-009-C8RA10314C-s001.pdf]

Electronic Supplementary Material (ESI) for RSC Advances.
This journal is © The Royal Society of Chemistry 2019

Supplementary information

Biomimetic channelled TiO_2/C catalyst with extremely low Pt loading for formaldehyde oxidation

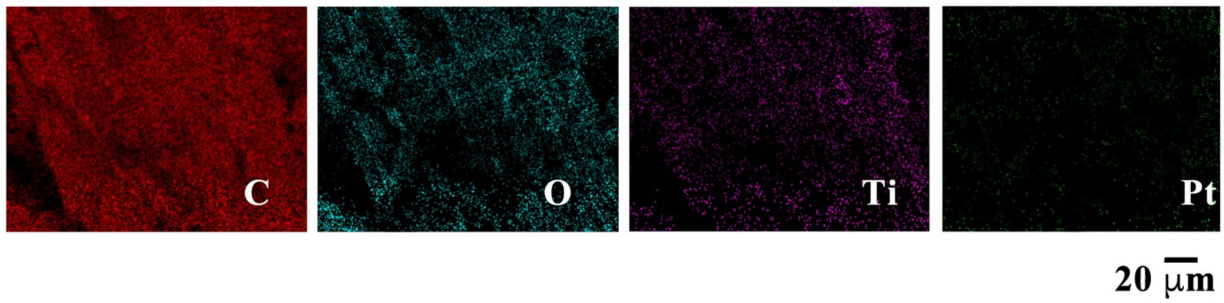

Figure S1. EDS elemental mapping of C, Ti, O, Pt for obtained TiO_2/C material (SEM image is shown in manuscript Fig.2 A).

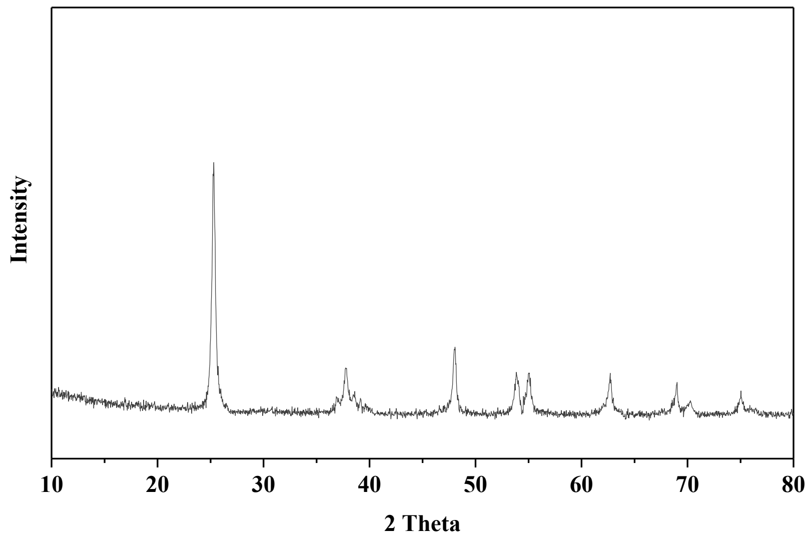

Figure S2. XRD pattern of sol-gel prepared TiO_2 without wood template. The result indicate the anatase phase of TiO_2.

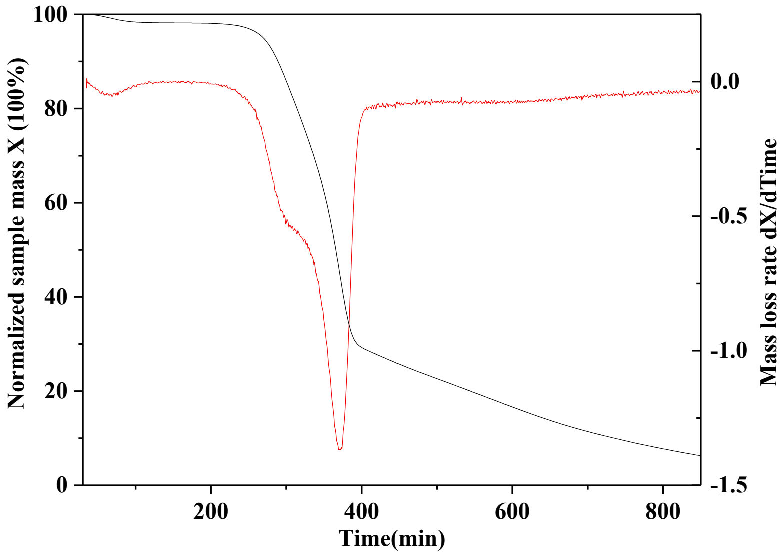

Figure S3. TGA curve of original wood chip without Ti and Pt precursor loading.

Table S1 Pt content determination in samples by ICP-MS

Samples*	Sample weight (mg)	Pt (mg)	Pt content (%)
#1(400°C)	15.2	0.014	0.092
#2(500°C)	12.8	0.012	0.095
#3(600°C)	11.5	0.011	0.098
#4(400°C-1)	18.5	0.012	0.063
#5(400°C-2)	25.1	0.010	0.041
#6(sol-gel method)	16.3	0.018	0.112

*The temperature noted in brackets are calcination temperature in the preparation.

Table S2 Surface area (S_{BET}), pore volume (V_{pore}) and pore size (d_{pore}) distribution of prepared porous TiO_2/C material

Temp. (°C)	S_{BET} (m^2 g^{-1})	V_{pore} (cm^3 g^{-1})	d_{pore} (nm)
400	41.5	0.055	2-10; 12-18
500	24.1	0.037	2-10
600	30.0	0.037	2-10
